# Short-term effects of an elimination diet and healthy diet in children with attention-deficit/hyperactivity disorder: a randomized-controlled trial

**DOI:** 10.1007/s00787-023-02256-y

**Published:** 2023-07-11

**Authors:** Annick Huberts-Bosch, Margreet Bierens, Verena Ly, Jessica van der Velde, Heleen de Boer, Gerry van Beek, Danielle Appelman, Sacha Visser, Lisa H. P. Bos, Lisa Reijmers, Jolanda van der Meer, Niki Kamphuis, Jos M. T. Draaisma, Rogier Donders, Gigi H. H. van de Loo-Neus, Pieter J. Hoekstra, Marco Bottelier, Alejandro Arias-Vasquez, Helen Klip, Jan K. Buitelaar, Saskia W. van den Berg, Nanda N. Rommelse

**Affiliations:** 1 Karakter, Child and Adolescent Psychiatry, Reinier Postlaan 12, 6525 GC Nijmegen, The Netherlands; 2 Institute of Psychology, Leiden Institute for Brain and Cognition, Leiden University, Leiden, The Netherlands; 3 Accare, Child and Adolescent Psychiatry, Groningen, The Netherlands; 4 Triversum—GGZ-NHN, Child and Adolescent Psychiatry, Alkmaar, The Netherlands; 5 Velp, The Netherlands; 6 Levvel, Center for Child and Adolescent Psychiatry, Amsterdam, The Netherlands; 7 Department of Pediatrics, Radboud Institute for Health Sciences, Radboud University Medical Center Amalia Children’s Hospital, Nijmegen, The Netherlands; 8 Department for Health Evidence, Radboud University Medical Center, Nijmegen, The Netherlands; 9 Department of Child and Adolescent Psychiatry and Accare Child Study Center, University of Groningen, University Medical Center Groningen, Groningen, The Netherlands; 10 Department of Psychiatry, Radboud University Medical Center, Nijmegen, The Netherlands; 11 Department of Human Genetics, Donders Institute for Brain, Cognition and Behavior, Radboud University Medical Center, Nijmegen, The Netherlands; 12 Department of Cognitive Neuroscience, Donders Institute for Brain, Cognition and Behavior, Radboud University Medical Center, Nijmegen, The Netherlands; 13 National Institute for Public Health and the Environment (RIVM), Bilthoven, Utrecht, The Netherlands

**Keywords:** ADHD, Dietary treatment, Child psychiatry, Emotion regulation

## Abstract

An Elimination Diet (ED) may be effective in reducing symptoms of Attention-Deficit/Hyperactivity Disorder (ADHD), but has never been compared to an active control condition [i.e., Healthy Diet (HD)]. In a two-armed RCT, a total of N = 165 children (5–12 years) with ADHD were randomized by means of minimization (1:1) to either an ED (*N* = 84) or HD (*N* = 81) within two Dutch child and adolescent psychiatry centers. The design included a non-randomized comparator arm including *N* = 58 children being treated with Care as Usual (CAU). Treatment allocation was unblinded. The primary outcome was a 5-point ordinal measure of respondership based on a combination of parent and teacher ratings on ADHD and emotion regulation, determined after 5 weeks of treatment. Ordinal regression analyses were done on an intention-to-treat basis. Fewer ED (35%) than HD (51%) participants showed a partial to full response, despite overall good-to-excellent treatment adherence (> 88%) and comparable high parental prior believes. A younger age and higher problem severity predicted a better respondership. CAU-preferring participants responded more often favorably (56%) compared to ED—but not HD—participants. Small-to-medium improvements in physical health (blood pressure, heart rate, and somatic complaints) were found in response to ED/HD versus decrements in response to CAU (74% received psychostimulants). The lack of superiority of the ED versus HD suggests that for the majority of children, dietary treatment response is not rooted in food-allergies/-sensitivities. The comparable results for treatment with HD and CAU are remarkable given that CAU participants were probably ‘easier to treat’ than HD (and ED) participants with proportionally fewer with a (suboptimal/non-response to) prior treatment with medication (4% versus 20%). Further assessment of long-term effects is needed to evaluate the potential place of dietary treatment within clinical guidelines. The trial is closed and registered in the Dutch trial registry, number NL5324 (https://www.onderzoekmetmensen.nl/en/trial/25997).

**Supplementary Information:**

The online version contains supplementary material available at 10.1007/s00787-023-02256-y.

## Introduction

The role of nutrition is an increasingly important research area in psychiatric disorders like Attention-Deficit/Hyperactivity Disorder (ADHD) which affects about 7.2% of children and adolescents [1]. A dietary approach frequently studied as potential intervention for ADHD is an elimination diet (ED) in which a limited number of foods are allowed to be consumed. Its rationale is that ADHD behaviors may be elicited by systemic adverse reactions to certain food allergens and potential food triggers. In fact, more than 70 years ago Bradley already made his first observations regarding the association between ADHD behaviors and allergic sensitivity in some children [2]. More recently, results of two meta-analyses and a recent review demonstrated that EDs may significantly reduce ADHD problems in about 30% of children [3–5]. Reductions in comorbid oppositional behaviors were also reported, potentially attributable to an improvement in emotion regulation [6].

Yet, despite these promising results and the long history of research, the topic remains controversial and clinical guidelines do not recommend EDs as a viable treatment for ADHD. The reservations concern large disparity in effect sizes, with results of studies with larger effects sizes not being replicated by independent research groups [7–9]. Furthermore, EDs require a full change of diet instead of omitting a few food items and are therefore difficult to study in a placebo-controlled manner. Consequently, several alternative explanations have been postulated for the superior effect of EDs compared (i.e., indirectly) to non-active control conditions that involved non-mandatory healthy diet advice with no constraints or obligations. Alternative explanations included changes in family or daily structure, parental treatment expectations, other non-specific treatment factors (e.g., contact with dietician and time investment), and non-specific improved nutritional quality and health.

The latter explanation is supported by observational studies, a systematic review and meta-analysis showing that unhealthy dietary patterns, high in total fat, saturated fat, refined sugars and grains, processed meats, sodium, and low in fruit and vegetables are associated with a higher risk of ADHD [10–14]. Such inadequate dietary patterns could lead to deficiencies in essential nutrients or higher intakes of certain food components (i.e., food additives) [15, 16], which might in turn adversely affect neurocognitive, behavioral, and physical development [15, 17, 18]. It is also possible that, on reverse, ADHD problems lead to unhealthy dietary behavior [10, 19, 20]. Preliminary results suggest that dietary interventions aimed at improving healthier food intake may reduce ADHD behaviors [21], although randomized-controlled studies are lacking.

Clearly, several vital clinical issues remain unanswered. Specifically, it is unknown whether EDs show superior effects when compared to an active control group with comparable impact on dietary constraints and similar non-specific factors such as parental treatment expectations. Most studies also have not addressed the feasibility of implementing dietary treatments in clinical practice. Finally, an understanding is still lacking of which factors might predict response to EDs.

To address these issues, the ‘Treatment of ADHD with Care as usual versus an Elimination diet’ (TRACE) study was carried out: a randomized head-to-head comparison of an ED and a Healthy Diet (HD) according to the Dutch dietary guidelines, thereby controlling for the impact of the diet and non-specific factors associated with following a diet (e.g., time investment, parental treatment expectations, contact with dietician, and daily structure). A non-randomized comparator arm was included with children receiving Care As Usual (CAU) to place results of the dietary treatments into context of the CAU effectiveness. The short-term (5-week treatment) effects in improving both ADHD and Emotion Regulation (ER) problems were examined, because one of the previous studies showed that the overall effect of an ED (a so-called Few Foods Diet) was clearer with > 57% scale reductions for behavioral/emotional impulsivity (hyperactivity-impulsivity, disruptive behavior) compared to inattentiveness [6]. ER problems also substantially contribute to impairment in children with ADHD [22].

## Method

### Study design

This study was a two-arm randomized-controlled trial (ED vs. HD) with a non-randomized comparator arm (CAU), performed within two child and adolescent psychiatric centers in the Netherlands: Karakter Child and Adolescent Psychiatry (Nijmegen) and Triversum—GGZ-NHN Child and Adolescent Psychiatry (Alkmaar). The study was approved by the local medical ethics Committee on Research involving Human Subjects (CMO), approval number: 2014-1349. The trial is registered in the Dutch trial registry, number NL5324. Treatment with CAU was added as a non-randomized comparator arm, because randomization of two dietary intervention versus CAU was not feasible [23]. Specifically, parents usually had a strong preference toward either a dietary intervention or CAU. This resulted in an extremely slow inclusion rate, a high number of drop-outs, and thereby unrepresentative groups of CAU. Therefore, the design was changed into a patient-preference design: parents could choose to participate in a dietary treatment (randomized) or in CAU. The CAU-preference group included children who started a new ADHD treatment (e.g., medication or psycho-education) within Karakter.

During the initial 5 weeks of the study, participants who received a dietary treatment were not allowed to start another treatment (e.g., medication or psychosocial intervention). If participants chose otherwise, this was coded as non-compliance. Medication treatment prior to the dietary treatment had to be discontinued no later than 1 week before the baseline assessment. CAU participants were not allowed to follow a strict dietary advice. If they chose otherwise, this was also coded as non-compliance. A detailed description of the procedures after five weeks can be found in the TRACE protocol paper [23].

### Participants

Participants were referred by clinicians or were recruited via advertisements and social media. Appendix S1 includes eligibility criteria. Next to the clinical ADHD diagnosis, an ADHD research diagnosis was established (Appendix S1). If the eligibility criteria were met, both parents (if applicable) filled out an informed consent. Children of 12 years old also signed this.

### Randomization and masking

Participants interested in a dietary intervention were randomized (1:1) to either an ED or HD with randomization within each participating center. Randomization by means of minimization was performed, including sex and age as factors, resulting in four groups. Within each group, blocked randomization was used (block size eight). An online program was used to generate the randomization sequence. Researchers enrolled participants and concealed the group allocation to parents via a sealed envelope. Participants, researchers, and dieticians were aware of treatment allocation after group allocation. It was impossible to allocate participants, researchers and dieticians blindly because of the complexity of the dietary treatments (e.g., it is impossible to be unaware of the limitations in food consumption).

### Procedures

Assessments took place at baseline before start of the dietary or CAU treatment (T0) and after 5 weeks of dietary or CAU treatment (T1). T0 was scheduled within 2 weeks prior to the start of the treatment.

#### Interventions

To facilitate adherence to the diets, parents received examples of menus, recipes, shopping lists, and advice for situations outside their home (e.g., parties). Parents also received a detailed list of which foods were allowed in which quantity and frequency. In both dietary treatments, weekly contacts with the dietician (via telephone or video calls) were scheduled. After 2 weeks, researchers contacted the family to evaluate experiences with the diet so far. Nutritional adequacy of the overall diet was continuously monitored and registered by the dietician. In both dietary treatments, care was taken by the dieticians to ensure that children did not lose weight.

##### Elimination diet

The goal of the ED was to exclude specific food components that could provoke ADHD and ER problems. The first part of the ED trajectory consisted of a 5-week elimination phase, where children followed a standardized ED supervised by a dietician. All known food allergens [proteins from milk, egg, wheat, fish (including shellfish and mollusks), soy, peanuts, and nuts] were eliminated and potential food triggers (gluten and histamine-releasing, or histamine-containing products) were reduced as much as possible. In addition, sugar intake was restricted in the elimination phase, because subjective reports of adverse effects of sugar are widespread, while consistent objective data are lacking [24, 25].

##### Healthy diet

The HD aimed to balance possible deficits in nutrient intakes or excessive intakes of nutrients, to improve ADHD and ER problems. This diet was based on the Dutch dietary guidelines of 2015 that were translated by The Netherlands Nutrition Centre into the recommended daily consumption of food groups per sex and age group [26, 27]. Consequently, some foods were allowed in unlimited quantities and frequencies (e.g., vegetables), others in restricted quantities and frequencies (e.g., chocolate sprinkles), some in very restricted quantities and frequencies (e.g., soft drinks), and some foods were not allowed (e.g., white bread). This HD was prescribed in a strict and structured manner, thereby making the diet comparable to ED regarding impact to the non-specific factors (e.g., time investment, daily structure). A detailed description of both dietary treatments can be found in the TRACE protocol paper [23].

##### Care as usual

According to the Dutch Multidisciplinary guidelines for the treatment of ADHD and authoritative international guidelines [28, 29], CAU for elementary school-aged children (5–12 years of age) consists of psycho-education, the prescription of medication approved for ADHD, and/or evidence-based parent, and/or teacher-administered behavior therapy, preferably both medication and behavior therapy. Appendix S2 describes which treatments the 58 CAU participants received.

### Outcomes

#### Demographics

The assessment of demographics is described in Appendix S3.

#### Primary outcome

The main outcome was response to treatment evaluated after 5 weeks on a 5-point ordinal measure of clinical respondership based on a combination of parent and teacher ratings of ADHD and ER [23]. A multi-informant-multi-dimensional compound score was created to optimally synthesize treatment effects and to reduce the need for multiple comparisons [30]. Parents and teachers were invited to rate the child’s ADHD problems of the past week, using the SWAN questionnaire, which was filled out online at T0 and T1. The SWAN consists of 18 DSM-IV-based items scored on a 7-point Likert scale ranging from 3 (*far below average*) to − 3 (*far above average*) with higher scores reflecting more ADHD problems [31]. Items 1–9 assess inattention problems and items 10–18 assess hyperactivity-impulsivity problems. Parents and teachers were also asked to fill out the Strengths and Difficulties Questionnaire (SDQ) at T0 and T1 to assess ER problems of the past week (using the SDQ Dysregulation Profile; SDQ-DP) [32]. The SDQ-DP includes 15 items representing emotional symptoms, conduct problems, and hyperactivity-inattention [33]. The items can be answered on a 3-point scale ranging from 0 (not true) to 2 (definitely true) with higher scores indicating more ER problems.

Response to treatment was evaluated by assessing the change in ADHD and ER problems at T0 and T1. A 30% or more symptom decrease was regarded as a significant beneficial response to treatment and a 30% or more symptom increase was regarded as significant deterioration of symptoms [34, 35]. The primary outcome variable ‘respondership’ was divided into five ordinal categories (see Appendix S4 for a detailed description):

1. Full responder: significant beneficial response on at least one parent *and* at least one teacher rated scale, and no significant deterioration on all scales.
2. Partial responder: significant beneficial response on at least one parent *or* at least one teacher rated scale, and no significant deterioration on all scales.
3. Mixed responder: significant beneficial response on at least one parent rated scale and significant deterioration on at least one teacher rated scale, or vice versa.
4. Non-responder: no significant beneficial response or deterioration on all scales.
5. Deterioration: significant deterioration on at least one parent or teacher rated scale, and no significant beneficial response on all scales.

The following measurements were taken into account to interpret the results of respondership (see Appendix S3 for a full description): food consumption of all treatment conditions at baseline, adherence to treatment, parents’ prior believes about the success and burden of treatment at baseline, time in weeks between start treatment and T1, total amount of time and consults needed during the dietician supervision, overall treatment trajectory experience, and Adverse Events (AE).

#### Secondary outcomes

Blood pressure, heart rate, height, body weight, Body Mass Index Standard Deviation Scores (BMI-SDS), sleep problems, and somatic complaints were assessed at T0 and T1 (see Appendix S3 for a full description). Additional secondary outcomes included emotional symptoms, conduct problems, peer-relationship problems, social behavior, family functioning, parenting styles, and carer-related quality of life (Appendix S3).

The number of questionnaires for CAU participants was reduced to compensate for not having a clear benefit of participating in contrast to participants being offered a dietary treatment, thereby enhancing CAU inclusion. Parents of CAU participants did not have to fill out questionnaires assessing family functioning, parenting styles, and carer-related quality of life. A total of 13 CAU participants only participated in measures that could be taken from home, due to time constraints. Consequently, data on IQ and physical measures were missing for these participants. This subsample did not differ from other CAU participants on relevant measures (Appendix S5).

### Statistical analyses

The justification of sample size is described in Appendix S6. All primary analyses were intention-to-treat and performed with SPSS (version 25). Differences on baseline characteristics between all treatment groups were determined with analysis of variance (ANOVA) and Chi-square analyses. A cumulative odds ordinal logistic regression was used to analyze the primary outcome (ordinal variable), based on the assumption that ED was superior to HD. The effect of the intervention was expressed in terms of odds ratio, comparing odds for reducing ADHD and ER problems in the ED group to the odds in the HD group. Proportions of respondership were compared between the treatment groups post hoc per category using a *z*-test with five Bonferroni corrections. Results of the dietary treatments were compared to results of the non-randomized CAU group.

Differences in continuous primary and secondary outcome measures between the treatments at T1 were determined with ANCOVA (the baseline value was added as covariate) and t-tests were used to assess within-group differences (T0 versus T1).

Ordinal logistic regression analyses and logistic regression analyses were used to assess baseline predictors for respondership and adherence to dietary treatment, with child and parent characteristics as predictors in addition to type of treatment (ED or HD). Only participants with good-to-excellent adherence to treatment were included in predicting respondership.

Explorative post hoc analyses and sensitivity analyses were used to further examine the effects found as well as to determine the influence of the COVID pandemic and a switch in parental raters between T0 and T1 on the results (supplementary material).

The impact of missing data was evaluated by comparing groups with and without unplanned and planned missing data on demographical data. Outliers were defined as values which were two standard deviations away from the mean. Outliers of secondary outcomes were replaced with the nearest value to the outlier. Correction for multiple comparisons was applied on secondary outcome measures using the false discovery rate (FDR) controlling procedure with a q value setting of 0.05 [36].

### Role of the funding source

The funders of the study monitored the progress of trial milestones and peer-reviewed the protocol as part of the grant awarding procedure. The corresponding and senior authors had full access to all the data in the study and had final responsibility for the decision to submit for publication.

## Results

**Fig. 1:**
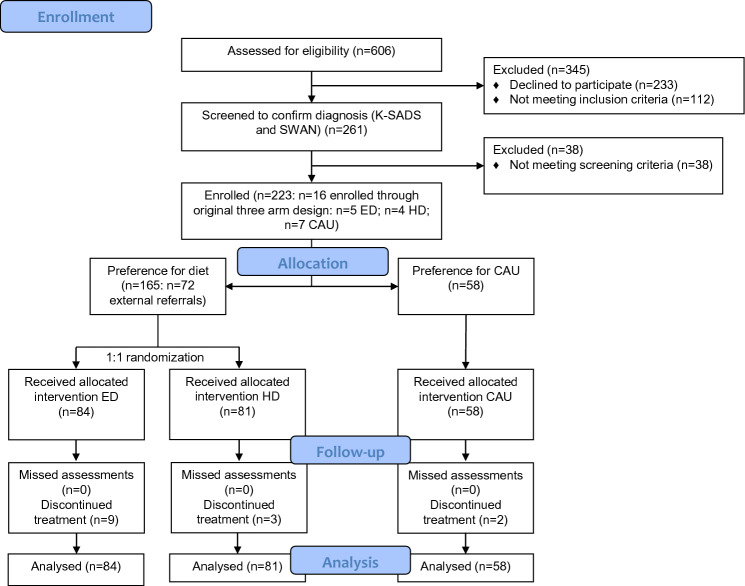
Trial profile

Between October 15, 2015 and March 31, 2021 (delay in recruitment due to the COVID pandemic), *N* = 165 patients were randomly assigned to receive the ED (*N* = 84) or the HD (*N* = 81). In addition, *N* = 58 participants were included in the non-randomized CAU-preferring arm (see Fig. 1 for the flowchart).

Table 1 describes baseline characteristics of children and parents per treatment group. No differences were found on the majority of characteristics between the dietary treatment groups, except for parental education (i.e., more highly educated mothers in the ED group (*p* = 0.004)). Compared to the non-randomized CAU group, a significant between-group difference was found on treatment history (i.e., less medication-naïve ED and HD participants compared to CAU-preferring participants (*p* = 0.023 and *p* = 0.011 respectively)).

**Table 1:** Baseline descriptive demographics

		Elimination diet	Healthy diet	Care as usual
		*N* = 84	*N* = 81	*N* = 58
		Mean (SD)	Mean (SD)	Mean (SD)
**Child**	Age at inclusion	8.52 (1.9)	8.82 (1.9)	8.27 (1.9)
	IQ	99.75 (13.8)	101.87 (11.3)	101.84 (15,1)
		% (N)	% (N)	% (N)
**Child**	Sex (male)	77.4 (65)	72.8 (59)	70.7 (41)
	ADHD presentation			
	Combined	56.0 (47)	56.8 (46)	56.9 (33)
	Inattentive^a^	34.5 (29)	30.9 (25)	29.3 (17)
	Hyperactive	3.6 (3)	3.7 (3)	10.3 (6)
	Not otherwise specified	6.0 (5)	8.6 (7)	3.4 (2)
	Comorbidities^b^			
	ODD (yes)^c^	36.9 (31)	44.4 (36)	34.5 (20)
	Probable ASD (yes)^d^	15.5 (13)	14.8 (12)	12.1 (7)
	Internalizing problems (yes)^e^	34.5 (29)	30.9 (25)	31.0 (18)
	Treatment history			
	Newly diagnosed	9.0 (15)	6.0 (10)	17.2 (10)
	Medication (yes)^f^	17.8 (27)	19.5 (29)	4.4 (4)
	Child focused therapy (yes)^g^	42.8 (65)	47.0 (70)	54.4 (49)
	Parent focused therapy (yes)^g^	27.0 (41)	25.5 (38)	28.9 (26)
**Mother**	Country of birth (The Netherlands)	92.9 (78)	95.1 (77)	86.2 (50)
	Employed (yes)	85.7 (72)	74.1 (60)	82.8 (48)
	Level of education			
	Primary/junior vocational	–	1.2 (1)	6.9 (4)
	Secondary^h^	72.6 (61)	87.7 (71)	74.1 (43)
	Higher professional/university	27.4 (23)	11.1 (9)	17.2 (10)
**Father**	Country of birth (The Netherlands)	86.9 (73)	91.4 (74)	93.1 (54)
	Employed (yes)	91.7 (77)	93.8 (76)	93.1 (54)
	Level of education			
	Primary/junior vocational	6.0 (5)	8.6 (7)	12.1 (7)
	Secondary^h^	78.6 (66)	81.5 (66)	70.7(41)
	Higher professional/university	15.5 (13)	9.9 (8)	15.5 (9)
**Parental psychopathology**	ADHD childhood^i^			
	Inattentive problems (yes)	12.0 (10)	24.7 (20)	20.3 (12)
	Hyperactive problems (yes)	9.6 (8)	16.0 (13)	11.9 (7)
	ADHD adulthood^j^			
	Inattentive problems (yes)	14.5 (12)	16.0 (13)	15.5 (9)
	Hyperactive problems (yes)	14.5 (12)	16.0 (13)	13.6 (8)
	Clinical level of psychological stress^k^	34.9 (29)	34.6 (28)	29. 8 (17)

*SD* standard deviation

^a^Appendix S7 shows demographics of the ADD presentation compared to the other presentations

^b^Appendix S8 describes how these variables were constructed

^c^Based on the K-SADS rated by parents and SDQ conduct problems subscale rated by teachers (see Appendix S8)

^d^Autism Spectrum Disorder: based on CSBQ and SDQ pro-social behavior subscale (see Appendix S8)

^e^Based on the SDQ emotional problems subscale, rated by parents and teachers (see Appendix S8)

^f^Including 1 HD participant with a treatment history of both stimulant and antipsychotic (aripiprazole) medication

^g^e.g., psycho-education or parental counseling

^h^Including: junior general secondary, senior secondary vocational, senior general secondary, and pre-university

^i^Using a cut-off score of ≥ 6 symptoms

^j^Using a cut-off score of ≥ 5 symptoms

^k^Based on the GHQ-12 using the cut-off score ≥ 3

Tables S4 and S5 (Appendix S9) show adherence to the dietary treatments based on dietician and parents’ scores and the percentage of agreement between both raters. Based on both raters, the majority of participants showed good-to-excellent adherence (91.9% of ED participants and 87.5% of HD participants). Chi-square tests revealed no differences between the two dietary treatment groups in adherence rated by parents (*χ*^*2*^ (2, *N* = 147) = 1.33, *p* = 0.515) and dietician (*χ*^*2*^ (2, *N* = 147) = 0.41, *p* = 0.813). A trend significant difference showed that almost three times as many ED participants (*N* = 9; 10.7%) quit the diet (but did not quit the study) before T1 compared to HD participants (*N* = 3; 3.7%), *χ*^*2*^ (1, *N* = 165) = 3.01, *p* = 0.083). These participants also showed more often insufficient adherence to treatment (until they quitted the diet) compared to participants who followed the diet until T1 (Appendix S9). Appendix S9 describes which factors predicted higher adherence to the diet: younger age, less severe emotion regulation problems at baseline rated by teachers, higher educational level of mothers, fathers’ country of birth the Netherlands, not often using the parenting style ‘punishment’, and higher parental prior believes about success of treatment.

Looking at other characteristics that were taken into account to interpret the primary outcome respondership (Appendix S9: Table S6), no differences were found on nutritional and health characteristics at baseline between the dietary treatment groups. Significantly more consults with the dietician were needed in the ED group than in the HD group (*p* = 0.012). In addition, parents in the ED and HD groups more often expected a relationship between food and child behavior compared to parents in the CAU-preference group (*p* < 0.0001). Moreover, parents in the CAU-preference group had higher parental prior believes about the success of treatment compared to parents in the HD group (*p* = 0.024). CAU participants showed lower energy (*p* = 0.029) and magnesium (*p* = 0.006) intake (the latter was non-significant after correcting for energy intake) compared to HD participants. Finally, AEs did not differ between the treatment groups, no serious AEs were reported, and no participants dropped out of the study before T1.

**Fig. 2:**
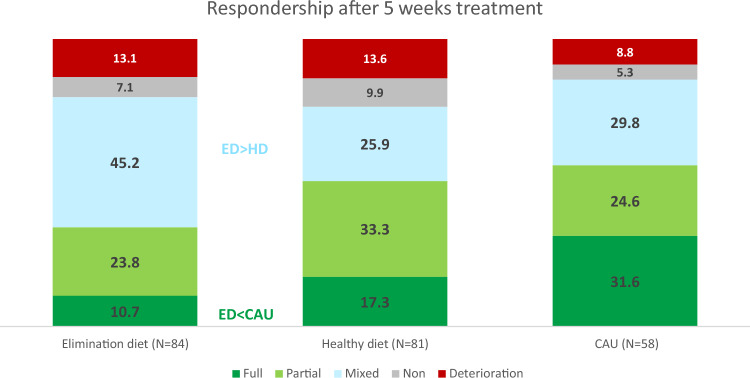
Distribution of respondership categories reflecting Change in ADHD and ER problems over time for the randomized ED and HD Groups and non-randomized CAU Group. Number represent %

Figure 2 displays the distribution of the respondership categories for all treatment groups. Assumptions of the cumulative odds ordinal logistic regression were met (Appendix S11). The odds ratio of being in a better respondership category for ED participants versus HD participants was 0.68, 95% CI [0.39, 1.19], *p* = 0.177, indicating that ED participants did not differ significantly from HD participants in terms of respondership. However, when proportions of respondership were compared post hoc per category, a significant difference was found for the category mixed respondership (Table S8 Appendix S12): more ED participants (45.2%) were categorized as mixed responders compared to HD participants (25.9%). The mixed responders in the ED group consisted more often of parents who report improvement and teachers who report deterioration compared to the HD (47.4% versus 38.1%, respectively) (Figure S1 in Appendix S13). No significant differences were found for the separate categories full and partial respondership, although combined significantly more HD participants (50.6%) were categorized as full or partial responders compared to ED participants (33.0%) (Table S11 Appendix S12).

The odds ratio of being in a better respondership category for the randomized ED group versus the non-randomized CAU group was 0.41, 95% CI [0.22, 0.76], *p* = 0.005, indicating that ED participants were less likely to end up in better respondership categories compared to CAU participants. Post hoc analyses to compare proportions of respondership showed that significant more CAU participants (31.6%) were categorized as full responders compared to ED participants (10.7%) (Table S9 Appendix S12). The odds ratio of being in a better response category for the randomized HD group versus the non-randomized CAU group was 0.62, 95% CI [0.33, 1.13], *p* = 0.119, and post hoc analyses showed no differences between proportions of respondership for HD versus CAU (Table S10 Appendix S12).

In addition, ANCOVA analyses were performed (Table 2). Most assumptions of ANCOVA were met (Appendix S11). No differences between the two main randomized dietary treatments at T1 were found in the single dimensional scores used for the composite primary outcome respondership category. Significant between-group differences at T1 between the dietary treatments and non-randomized CAU group were found (medium-to-large effect sizes; range 0.13–0.15): CAU was associated with lower inattention, hyperactivity-impulsivity, and emotion regulation problems reported by teachers after 5 weeks of intervention compared to both dietary treatments. Within-group differences showed a decrease in inattention, hyperactivity-impulsivity, and emotion regulation problems reported by parents after 5 weeks in all treatment groups with medium-to-large effect sizes (range 0.53–0.82) in the dietary treatments and small-to-medium effect sizes (range 0.41–0.68) in the CAU group. A decrease in the same problem behaviors was also reported by teachers after 5 weeks in the HD (small effect sizes; range 0.23–0.34) and CAU (large effect sizes; range 0.81–1.00) groups, but not ED.

**Table 2:** Results of ANCOVA of single dimensional scores used for the composite primary outcome respondership

	Elimination diet				Healthy diet				Care as usual				Between-group differences T0	Between-group differences T1		
	*N* = 84				*N* = 81				*N* = 58							
	T0	T1	T0–T1		T0	T1	T0–T1		T0	T1	T0–T1					
	M (SD)	M (SD)	d	*p*-value	M (SD)	M (SD)	d	*p*-value	M (SD)	M (SD)	d	*p*-value	*p*-value	F	*p*-value	ƞ_p_^2^
Parent ratings (*N* = 160; 97.0%)																
Inattention^a^	1.5 (0.7)	1.0 (0.9)	0.66	< 0.0001	1.3 (0.6)	1.0 (0.9)	0.53	< 0.0001	1.3 (0.6)	1.1 (0.8)	0.41	0.003	0.133	1.05	0.352	0.10
Hyperactivity-impulsivity^a^	1.4 (0.6)	0.9 (0.8)	0.73	< 0.0001	1.4 (0.8)	1.0 (0.8)	0.61	< 0.0001	1.2 (0.9)	0.9 (0.8)	0.54	< 0.0001	0.411	0.81	0.447	0.01
Emotion regulation^b^	1.0 (0.3)	0.8 (0.3)	0.82	< 0.0001	0.9 (0.3)	0.8 (0.3)	0.66	< 0.0001	0.9 (0.3)	0.8 (0.3)	0.68	< 0.0001	0.166	1.80	0.167	0.17
Teacher ratings (N = 153; 92.7%)																
Inattention^a^	1.3 (0.8)	1.3 (0.8)	0.05	0.656	1.3 (0.7)	1.2 (0.7)	0.29	0.016	1.1 (0.8)	0.6 (0.9)	1.0	< 0.0001	0.195	17.86	ED = HD < CAU *p* < 0.0001	0.15
Hyperactivity-impulsivity^a^	1.2 (1.0)	1.2 (1.0)	− 0.06	0.597	1.4 (0.8)	1.2 (0.9)	0.34	0.005	1.2 (1.0)	0.7 (0.9)	0.81	< 0.0001	0.341	14.80	ED = HD < CAU *p* < 0.0001	0.13
Emotion regulation^b^	0.8 (0.3)	0.8 (0.3)	0.03	0.818	0.9 (0.3)	0.8 (0.3)	0.23	0.051	0.8 (0.3)	0.6 (0.3)	0.86	< 0.0001	0.381	15.55	ED = HD < CAU *p* < 0.0001	0.13

M (SD); Values represent unadjusted mean (standard deviation); d = Cohen’s d; ƞ_p_^2^ = partial eta squared; ^a^ Range 3 to − 3 (higher scores reflect more symptoms); ^b^ Range 0–2 (higher scores reflect more symptoms)

Ordinal logistic regression analyses were run to determine which child or parental factors could predict respondership taking into account type of dietary treatment. Results showed that lower parental quality of life (OR 0.20, 95% CI [0.08, 0.51], *p* = 0.001) and higher parental stress (OR 0.27, 95% CI [0.12, 0.62], *p* = 0.002) at baseline predicted worse response to the dietary treatments. No significant interaction effects were found between both predictors and treatment in predicting respondership (OR 0.49, 95% CI [0.14, 2.22], *p* = 0.353) and (OR 0.30, 95% CI [0.07, 1.27], *p* = 0.101), respectively. Sugar intake at baseline was elevated in all groups and normalized during the dietary treatment, but this did not predict response to treatment. Post hoc non-planned comparisons were performed to determine which factors could predict specific respondership categories (Appendix S14).

**Fig. 3:**
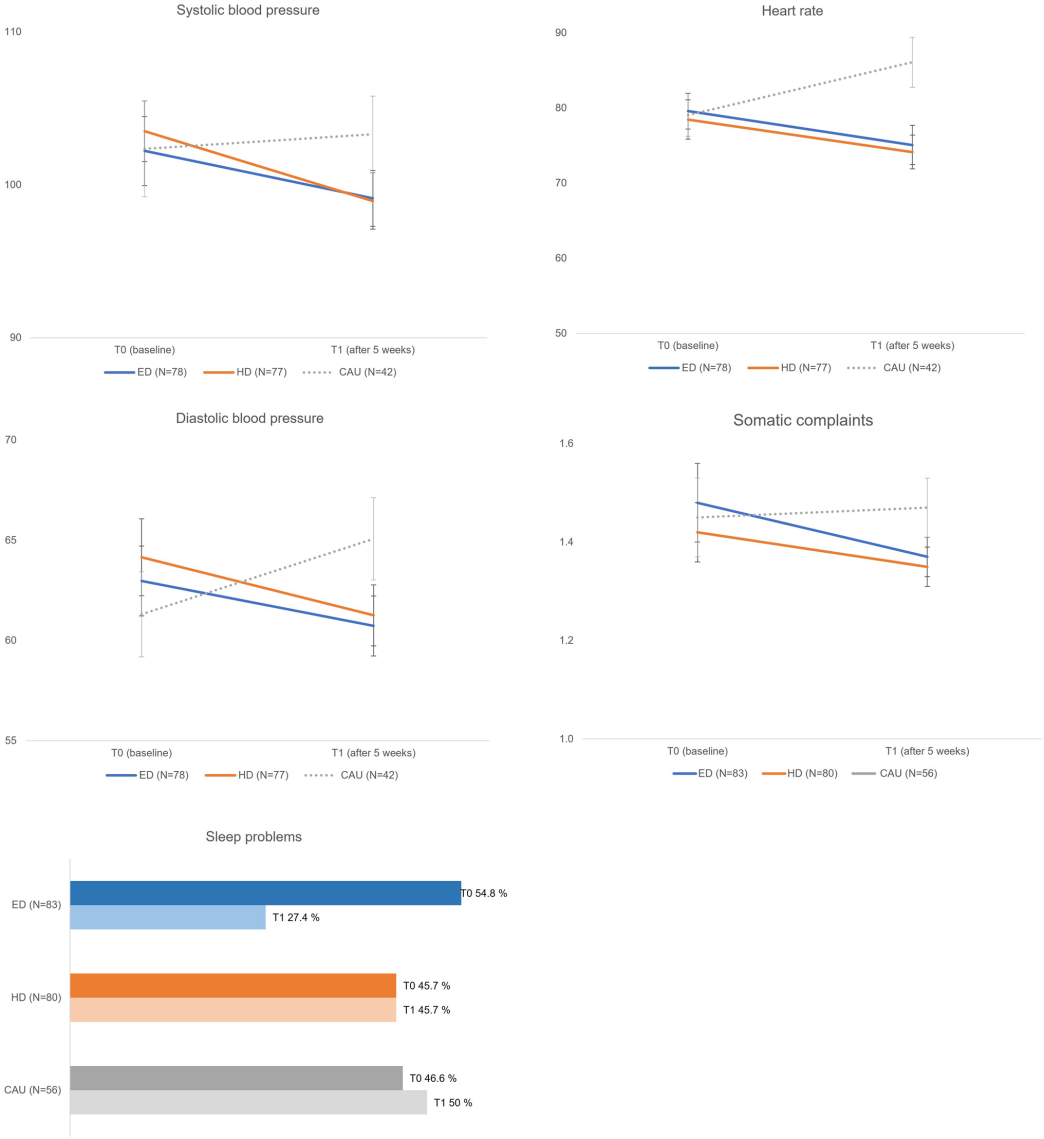
Results of ANCOVA of heart rate, blood pressure, sleep problems and somatic complaints. Effect sizes for between and within differences (ƞ^2^ and Cohen’s d, respectively) are depicted for heart rate, blood pressure, and somatic complaints. This was not applicable for the categorical variable sleep problems

Table S15 (Appendix S15) illustrates the between-group differences at T0 and T1 and within-group differences over time for the secondary outcomes, adjusted for baseline scores. There were no between-group differences between groups at T0. This demonstrates that the CAU-preference group was largely comparable to the dietary treatments in terms of child and parents characteristics (Table S15 Appendix S15). There were no significant between-group differences at T1 between the two randomized dietary treatments. There were several significant between-group differences at T1 between the dietary treatments and the non-randomized CAU group (Fig. 3): heart rate, systolic and diastolic blood pressure, and somatic complaints (e.g., stomach ache) of participants in both dietary treatments decreased more over time compared to the CAU group. In addition, sleep problems decreased more in the ED group over time compared to the CAU group.

Within-group differences (Table S15 Appendix S15) in the ED group showed that mean BMI-SDS slightly decreased, due to a small decrease in mean body weight (0.67 kg) and a small increase in mean length of 0.49 cm (note that mean BMI at T1 was still above the median). In addition, mean heart rate, systolic blood pressure, diastolic blood pressure, sleep problems, somatic complaints, and parental stress decreased, and parental happiness and quality of life increased over time. The same within-group differences were found for the HD group, except for parental happiness and sleep problems. In the CAU group, mean heart rate and diastolic blood pressure increased over time and parental stress decreased over time.

Different sensitivity analyses were run. First, a sensitivity analysis without participants that quit the diet before T1 was performed, to examine if the results of the ordinal regression analysis changed. Results showed the same pattern: the odds ratio of being in a better response category for ED participants versus HD participants was 0.74, 95% CI [0.42, 1.31], *p* = 0.294. Second, sensitivity analyses indicated no relevant differences between groups with or without (un)planned missing data (Appendix S2). Finally, a switch in parental raters between T0 and T1 and the COVID pandemic did not influence the main results of the primary outcome (Appendix S16 and S17, respectively).

## Discussion

Fewer ED (35%) than HD (51%) participants showed a partial to full response, despite overall good-to-excellent treatment adherence (> 88%) and comparable high parental prior believes. A younger age and higher problem severity predicted a better respondership. CAU-preferring participants responded more often favorably (56%) compared to ED—but not HD—participants. Small-to-medium improvements in physical health (blood pressure, heart rate, and somatic complaints) were found in response to ED/HD versus decrements in response to CAU (74% received psychostimulants). The lack of superiority of the ED versus HD suggests that for the majority of children, ADHD problems are not rooted in food-allergies/-sensitivities. The comparable results for treatment with HD and CAU are remarkable given that CAU participants were probably ‘easier to treat’ than HD (and ED) participants with proportionally fewer with a suboptimal/non-response to prior treatment with medication (4% versus 20%). Further assessment of long-term effects is needed to evaluate the potential place of dietary treatment within clinical guidelines.

The finding that an ED is not more effective than an HD may be seen as surprising given results of the largest previous RCT comparing an ED (the Few Foods Diet) to receiving healthy food advice without active guidance by a dietician [6]. The ED used in the current study is the ‘standard operating procedure’ for (restricted) EDs; diets used to attempt to diagnose and treat food-allergies and intolerances toward natural and/or artificial ingredients [5]. In line with expectations, the proportion of responders in the ED was comparable to previous studies (~ 33%) [5]. Furthermore, even though a trend significant difference showed that somewhat more ED participants quit the diet before T1 compared to HD participants, analyses excluding these participants gave similar results. Finally, it seems unlikely that child or parental characteristics may play a role, as randomization was used to allocate treatment and both groups did not differ on a broad set of measures at baseline including treatment expectancy. Therefore, the lack of superiority of the ED versus HD suggests that a relationship between food intake and ADHD is for the majority of children not rooted in an allergic/overreactive response to food ingredients. Rather, a suboptimal nutritional quality, restored with the HD, may luxate and/or aggravate ADHD and related behaviors in a proportion of children.

In almost half of the children within the ED group, parents reported beneficial response to treatment and teachers reported deterioration. This pattern was not seen in the HD or CAU group. In previous ED studies, effects observed by parents were reduced when ratings of teachers were taken into account [4, 5, 38]. Only one study discovered effects observed by both parents and teachers [6]. These different views of raters might be explained by the large parental investments necessary to apply an ED [15]. Time investment by dieticians was the same for both dietary treatments; however, more consults were needed in the ED group compared to the HD group. Specifically, parents in the ED group more often needed guidance (e.g., about which foods or ingredients were allowed) next to the weekly appointments in applying the diet in daily life. Taking these higher levels of parental effort into account, parents could have had an investment in the ED being a success [4]. Another explanation might be that parents in the ED group had higher expectations of benefits of treatment, despite the fact that we did not observe differences in prior believes between groups (this might be due to a ceiling effect). This is supported by a systematic review and meta-analysis showing that different contextual and psychological factors (such as expectation of benefit) not related to the treatment (medication in that case) may explain response to treatment [39]. Finally, although not systematically assessed, one of the success factors for adherence reported by parents was participation of the whole family; the HD is probably more suitable for this than the ED.

Next to establishing efficacy of the dietary treatments, we assessed which children may benefit the most from a dietary treatment. Previous studies showed that no one factor stands out in predicting response to both pharmacological and nonpharmacological treatment [40]. Results of the present study indicate that children with less severe inattention and emotion regulation problems have higher chances of showing ambiguous dietary effects (i.e., mixed responders). Together with factors that predict good adherence, this could mean that indicators for benefiting from and choosing to follow a dietary treatment in clinical practice include a younger age of children and a high severity of problems in different contexts (i.e., at school and at home) and in multiple areas including inattention, hyperactivity, impulsivity, and/or emotion regulation problems. The latter, however, may complicate adherence to treatment. In addition, parents need to have faith in the possible efficacy of dietary treatments. Contra-indicators include less parental mental resources beforehand (i.e., parents with higher stress levels, lower quality of life, using more punishments in the upbringing, or parents with a first-generation migration background). These factors might need attention before starting a dietary treatment.

Compared to CAU participants, dietary treatment participants performed better on physical measurements after 5 weeks. This suggests that dietary treatment participants improve more than CAU participants on symptoms that usually accompany the disorder, such as sleep problems and somatic complaints (e.g., bowel problems) [41, 42]. Possibly, these improvements might in turn lead to a decrease in ADHD and ER problems in the long term [41, 42]. On the other hand, CAU participants were more likely to end up in a better response category than the ED participants after 5 weeks. This may be caused by the fact that response to medication is rather fast in most cases: an example is shown in an exploratory study on the efficacy of Omega-3/6 fatty acids [43], where eventually scale scores in the Omega-3/6 group leveled off to almost the same level as CAU after a longer time period. Therefore, long-term effects are needed to examine if the ED (and HD) results might also change over a longer time period and if the effects level off toward the CAU level. Another possible explanation for the better response in CAU compared to ED is that more ED participants (17.8%) had medication as treatment history compared to the CAU participants (4.4%). This is also higher compared to a previous dietary treatment study with 12% of children who received treatment with psychostimulants [6]. These pretreated children might be more difficult to treat [44], since this group mostly did not respond well to pharmacotherapy, which might suppress the possible effects of a dietary treatment. However, since the CAU arm was not randomized, above-described results should be interpreted with caution.

Results also revealed that parents in the dietary treatment groups more often expected a relationship between food and child behavior than parents in the CAU group. Also, baseline measurements of nutritional intake showed a slightly higher intake of different beneficial macro- and micronutrients in the dietary treatments compared to the CAU group. This could indicate that parents in the dietary treatments were more aware of the importance of healthy dietary patterns compared to CAU. If positive effects are already found in nutrition-oriented families, the effects might be even larger in families who are not yet nutrition-oriented. However, CAU-preferring participants did not choose to follow a dietary treatment for several reasons (e.g., time investment). Therefore, offering a less invasive HD instead of an ED might be an outcome for these families.

A strength of the study was the randomized comparison of two active dietary treatment approaches, with similar impact on household rules, the amount of structure offered to children, and the amount of time investment by the dietician. Furthermore, we conducted the study within mental health care facilities that also deliver CAU and used few inclusion and exclusion criteria. This contributes to the generalizability of the results to a broader population of children with ADHD and provides a more balanced view on the clinical utility of dietary treatments. Other strengths include the large sample size, an overall good-to-excellent adherence to the diets, including both parent and teacher ratings in the primary outcome, as well as examining effects on a wide range of secondary outcomes such as physical measurements and parental well-being, and including participants from different parts of the Netherlands, thereby enhancing generalizability of the results.

The study has some limitations. First, parents were not blinded to treatment allocation. Despite of this, prior believes about the effects of treatment did not differ between the two dietary treatment groups at baseline, suggesting that this did not influence effects of treatment. Second, the original three-arm randomized-controlled trial seemed not to be feasible and was changed into a two-arm randomized-controlled trial (ED vs. HD) with a non-randomized comparator arm (CAU). Consequently, the CAU group could only be used as a reference group.

## Conclusion

All in all, considering the lower number of mixed responders in the HD group (i.e., less ambiguous effects), the lower number of HD participants who quit the diet, and previous studies showing beneficial effects of the HD in children with ADHD [10, 15, 21], HD might be considered in ADHD care as a starter or as co-treatment supervised by a dietician for motivated parents who are interested in dietary treatments and with enough mental resources beforehand. Despite the promising results, longer follow-up studies are needed to examine whether the short-term results change over time; the feasibility of adhering to a diet over a longer period of time and the high burden this may place on families; and the potential risks of nutritional deficiencies in the long term. Therefore, the TRACE study will follow up on the participants after 4, 8, and 12 months [23].

### Supplementary Information

Below is the link to the electronic supplementary material.

Supplementary file1 (JPG 582 KB)

Supplementary file2 (PDF 479 KB)

## Data Availability

Researchers may request access to individual participant data or analytic code by providing a proposal. Proposals should be directed to a.bosch@karakter.com; to gain access, data requestors will need to sign a data access agreement. The study protocol and informed consent forms are available online [23].
