# Supplementary figures and images for: Short-term effects of an elimination diet and healthy diet in children with attention-deficit/hyperactivity disorder: a randomized-controlled trial
Source: Eur Child Adolesc Psychiatry. 2023 Jul 11;33(5):1503–16. doi: 10.1007/s00787-023-02256-y (PMC11098970; doi:10.1007/s00787-023-02256-y)

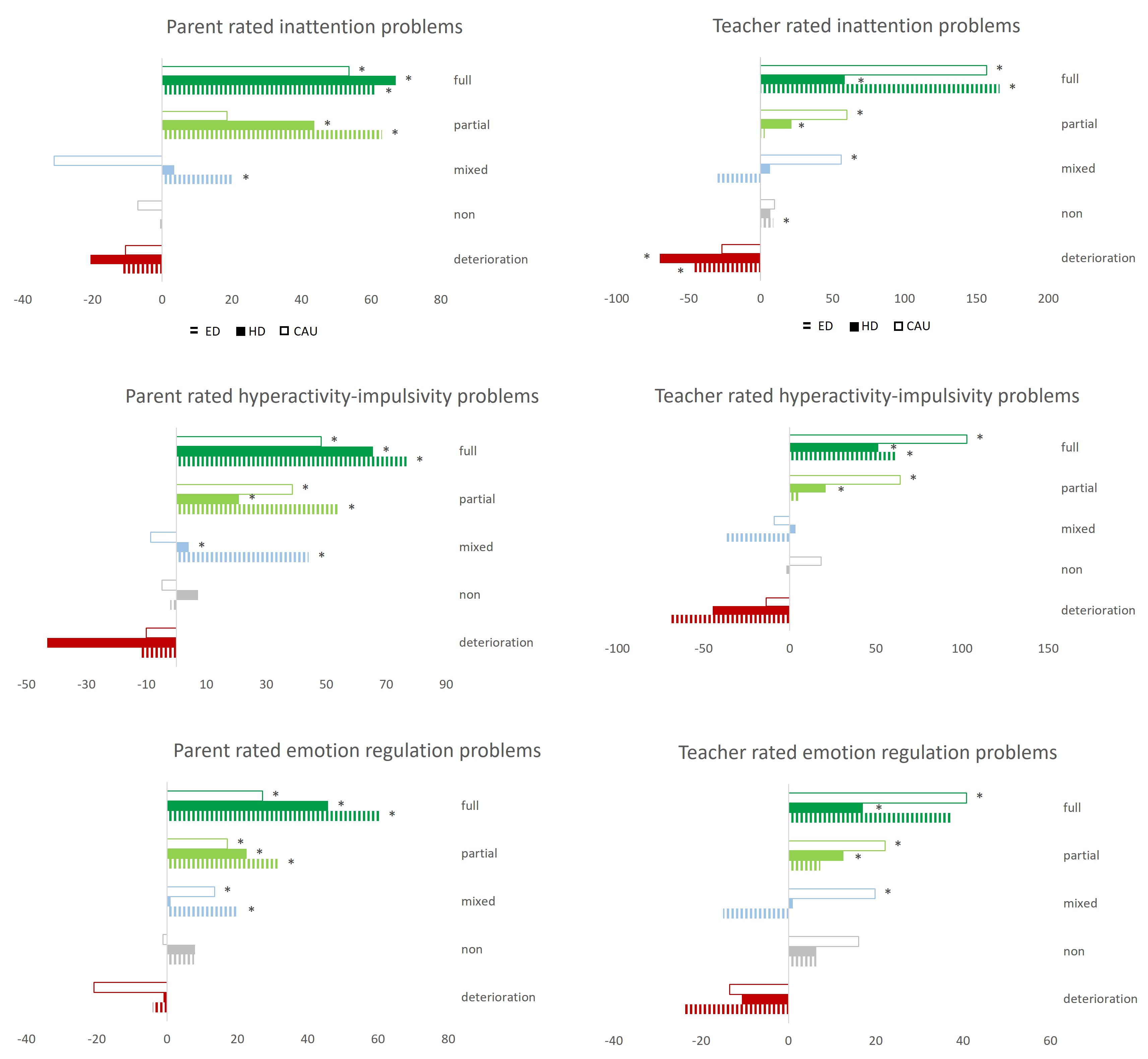

Supplement: Supplementary file 1 — Supplementary file1 (JPG 582 KB) [file 787_2023_2256_MOESM1_ESM.jpg]
