# Supplementary material for: Short-term effects of an elimination diet and healthy diet in children with attention-deficit/hyperactivity disorder: a randomized-controlled trial
Source: Eur Child Adolesc Psychiatry. 2023 Jul 11;33(5):1503–16. doi: 10.1007/s00787-023-02256-y (PMC11098970; doi:10.1007/s00787-023-02256-y)
Supplement: Supplementary file 2 — Supplementary file2 (PDF 479 KB) [file 787_2023_2256_MOESM2_ESM.pdf]

Supplementary material

Table of contents

**Supplement**	**Pages**
S1: inclusion & exclusion criteria and research diagnosis	2
S2: CAU treatment	3
S3: description outcome measures	4-5
S4: responderhip	6-7
S5: (un)planned missing data	8
S6: sample size calculation	9
S7: ADD presentation	10-11
S8: assessment of comorbidity	12
S9: measurements that were taken into account to interpret the results of respondership	13-16
S10: micronutrient intake	17
S11: assumptions statistical analyses	18
S12: comparisons of proportions of respondership	19-21
S13: percentage change in T0 versus T1	22-23
S14: predicting specific respondership categories	24-25
S15: results of ANCOVA of secondary outcomes	26-27
S16: different parental raters at T0 and T1	28
S17: influence Covid-19	29
References	30-31

Supplement S1: inclusion & exclusion criteria and research diagnosis

For eligibility, participants had to meet the following criteria: clinical and research ADHD diagnosis according to the DSM-5 (any presentation) and 5-12 years old at the inclusion date. Comorbidities were allowed except for eating disorders (i.e. anorexia or bulimia nervosa) and diabetes mellitus. Exclusion criteria were insufficient mastery of Dutch language in parents or children; current treatment for ADHD that could not be discontinued or was not stabilized; severe parent-child relationship problems requiring family therapy; unwillingness to have meat or animal food products in the diet (without these products it is impossible to achieve nutritional adequacy of the overall diet for ED participants). Two participants (one HD and one ED participant) continued using a stable dosage of risperidone during the diet. Discontinuation of risperidone was not advisable for these participants.

Next to the clinical ADHD diagnosis, an ADHD research diagnosis was established (Supplement A). based on the Kiddie Schedule for Affective Disorders and Schizophrenia (K-SADS) [1] in combination with teacher reports on the Strengths and Weaknesses of ADHD-symptoms and Normal-behaviors rating scale (SWAN) [2, 3]. For six participants, the screening was incomplete (e.g. K-SADS and/or SWAN missing). For these participants, the clinical ADHD DSM-5 diagnosis was used to classify the ADHD presentation.

Supplement S2: CAU treatment

Table S1. Overview of Types of Treatment received by the CAU Participants

	% (N)
**One type of treatment**	
Medication	65.5 (38)
Parental group behavioral therapy	1.7 (1)
Parental guidance	1.7 (1)
Intensive home treatment	5.2 (3)
Psycho-education parents	3.4 (2)
Psycho-education child	10.3 (6)
**Combination of treatments**	
Medication and psycho-education parents	1.7 (1)
Medication and cognitive behavioral therapy	1.7 (1)
Medication and intensive home treatment	5.2 (3)
Parental guidance and psycho-education for both parents and child	1.7 (1)
Psycho-education for child and parents	1.7 (1)

Supplement S3: description outcome measures

Demographics

Descriptive measures at T0 included 1) total IQ estimated using five subtests of the Wechsler Intelligence Scale for Children (WISC-III) [4]; 2) demographics (e.g. SES), and 3) self-reported parental psychopathology using the ADHD rating scale (46 items assessing ADHD symptoms of the past six months and of the ages between 0 and 12 years old) [5] and the General Health Questionnaire (GHQ-12; 12 items assessing general health of the past week) [6].

Secondary outcomes

Body Mass Index Standard Deviation Scores (BMI-SDS) were calculated using the Dutch growth reference data [7, 8]. Sleep problems were examined using a 5-item questionnaire assessing problems with falling asleep, sleeping through, and total amount of sleep compared to peers of the past week. Somatic complaints, of the past week, were assessed using the Pittsburgh side-effects rating scale (18 items) [9].

Other secondary outcomes included emotional symptoms, conduct problems, peer relationship problems, and social behaviors of the past week: parents and teachers were asked to complete the Children's Social Behavior Questionnaire (CSBQ; 40 items) [10] and the SDQ (25 items) [11]. These were all completed at both T0 and T1. Family functioning and parenting styles, of the past week, were assessed using the Family Functioning Questionnaire including 28 items (FFQ: English translation of 'Vragenlijst Gezinsfunctioneren Ouders (VGFO)') [12] and the Brief Scale of Parental Behavior including 25 items (BSPB: English translation of 'Verkorte Schaal voor Ouderlijk Gedrag (VSOG)') [13]. The CarerQol instrument (7 items) [14] and the Parenting Stress Questionnaire including 34 items (PSQ: English translation of 'Opvoedingsbelasting Vragenlijst (OBVL)') [15] measured carer-related quality of life in caregivers in the current situation. These were all completed at both T0 and T1.

Measurements that were taken into account to interpret the results of respondership

Food consumption (all treatment conditions) was measured through an online tool ('Eetmeter', Dutch Nutrition Center) available at the website of the Dutch Nutrition Center or as a mobile app (free of charge) [3]. The validity of this tool is sufficient [16]. Parents were asked to report all food consumed by the child, for two weekdays and one weekend day before T0 and send this information to the research staff by email (an export function is part of the online tool). The exported files included macro- and micronutrient values originated from the Dutch Food Composition Database (NEVO) [17] and the Dutch Nutrition Center Database for all reported foods. Based on this information, mean daily nutrient intake was calculated. Adherence to treatment was assessed on a 10-point scale (ranging from 1 no adherence to 10 perfect adherence to the diet) by dieticians and parents separately every week. An aggregated adherence measure was created: 1) excellent (i.e. every week scores of eight or higher);

2) good (i.e. scores not lower than six) and 3) insufficient (i.e. at least once a score lower than six). Adherence was only calculated for participants with at least two weeks of adherence data. Parents' prior believes about the success and burden of treatment were evaluated using a 5-item questionnaire at T0 and T1. Time in weeks between start treatment and T1 was calculated and total amount of time and consults needed during the dietician supervision was calculated. Parents rated their overall treatment trajectory experience on a scale of 0 to 10 using the GGZ-Thermometer, with higher scores reflecting more satisfaction with the treatment trajectory (http://www.ggznederland.nl/leden/thermometer/handleiding.html). Adverse events (AE) were assessed, which is described in the TRACE protocol paper [3].

Supplement S4: respondership

Response to treatment was evaluated by assessing the change in ADHD and emotion regulation problems of the past week at T0 and T1 (i.e. (T0-T1)/ T0 * 100) [18]. Two exceptions to this formula were included: 1) if the T0 score was zero, no change score could be computed. Therefore, value one was added to the T0 and T1 score to be able to compute a change score; 2) absolute values were used to ensure that an improvement was not coded as deterioration or vice versa. A 30% or more symptom decrease was regarded as a significant response to treatment and a 30% or more symptom increase was regarded as significant deterioration of symptoms. The primary outcome variable 'respondership' is divided into five categories:

1. Full responder (significant response on both parent and teacher rated scales):
    a. ≥ 30% improvement on at least one of three parent rated scales AND ≥ 30% improvement on at least one of three teacher rated scales AND on none of the parent and teachers scales ≥ 20% deterioration
    b. OR: ≥ 30% improvement including one teacher rated scale and two parent rated scales or vice versa AND on maximally one scale a deterioration between 20% and 25% AND on all other scales a maximum deterioration of ≤ 20%
2. Partial responder (significant response on parent or teacher rated scale):
    a. ≥ 30% improvement on at least one of three parent rated scales AND on all three teacher scales no improvement of ≥ 30% AND all scales a maximum deterioration of < 30%
    b. OR: ≥ 30% improvement on at least one of three teacher rated scales AND on all three parent scales no improvement of ≥ 30% AND all scales a maximum deterioration of < 30%
    c. OR: improvement between 20% and 30% on at least one of three parent rated scales AND improvement between 20% and 30% on at least one of three teacher rated scales AND all scales a maximum deterioration of < 30%
    d. OR: ≥ 30% improvement on at least one of three parent rated scales AND ≥ 30% improvement on at least one of three teacher rated scales AND one scale a deterioration between 25% and 30% AND all other scales a maximum deterioration of < 30%
3. Mixed responder (significant response on at least one parent rated scale and significant deterioration on at least one teacher rated scale or vice versa, or a significant difference within rater):
    a. ≥ 30% improvement on at least one of three parent rated scales AND ≥ 30% deterioration on at least one of three teacher scales
    b. OR: ≥ 30% improvement on at least one of three teacher rated scales AND ≥ 30% deterioration on at least one of three parent scales

c. OR: ≥ 30% improvement on at least one of three parent rated scales AND ≥ 30% deterioration on at least one of three parent scales
d. OR: ≥ 30% improvement on at least one of three teacher rated scales AND ≥ 30% deterioration on at least one of three teacher scales

4. Non-responder (no significant response): all six scales show no ≥ 30% improvement or ≥ 30% deterioration
5. Deterioration (significant deterioration on at least one parent or teacher rated scale): ≥ 30% deterioration on at least one of three parent rated scales OR ≥ 30% deterioration on at least one of three teacher rated scales AND a maximum improvement of < 30% on all scales

Supplement S5: (un)planned missing date

Planned missing data

For 13 CAU participants data was missing, because these participants chose to participate only in the measures that could be taken from home. This resulted in planned missing data for IQ and physical measurements (blood pressure, heart rate, weight, height). Analyses were performed to examine if this subsample of CAU participants differed on demographical data (see Table 1 for an overview of the demographical data) compared to the other CAU participants. One statistically significant between group difference was found: parents in the former group had higher prior believes about success of treatment (*M* = 3.33, *SD* = 0.61) compared to parents in the latter group (*M* = 3.81, *SD* = 0.50), ($t(219) = -2.76$, $p = 0.006$).

Unplanned missing data

For 23 participants (N = 7 ED participants; N = 10 HD participants; N = 6 CAU participants), there was unplanned missing data of parents or teachers on the primary outcome measures. Consequently, these participants could not be categorized in respondership categories in which data of both raters was needed (i.e. full or mixed responders). Analyses were performed to examine if this subsample differed on demographical data compared to participants without these missing data. Two statistically significant between group differences were found. First, fathers in the former group more often had another country of birth than the Netherlands (21.7%) compared to fathers in the latter group (8.0%), $\chi^2(1) = 4.52$, $p = 0.034$. Second, parents in the former group experienced less often clinical levels of stress (9.1%; based on the GHQ-12) compared to parents in the latter group (36.0%), $\chi^2(1) = 6.46$, $p = 0.011$.

Supplement S6: sample size calculation

The justification of sample size was calculated based on the assumption of superiority, i.e. that ED was more effective than the HD on the ordinal primary outcome respondership (i.e. five categories). A clinically relevant outcome was defined as detecting twice as many full responders in the ED group than in the HD group. Each dietary group included 81 children. With this sample size and using ordinal regression, the power was 0.99 ($\alpha = 0.05$, two-sided test) to detect double the amount of full responders in the ED compared to the HD (Table S1: scenario 1). In addition, the power to detect one and a half times as many full responders in the ED compared to the HD was 0.64 ($\alpha = 0.05$, two-sided test) (Table S1: scenario 2).

Table S2: Hypothetical Distribution of Participants for Power Calculation

	Scenario 1				Scenario 2			
	Elimination Diet N = 81		Healthy Diet N = 81		Elimination Diet N = 81		Healthy Diet N = 81	
	N	%	N	%	N	%	N	%
Full responder	16	20%	8	10%	12	15%	8	10%
Partial responder	45	55%	24	30%	37	45%	24	30%
Mixed responder	10	12%	21	25%	13	16%	21	25%
Non-responder	6	8%	16	20%	11	14%	16	20%
Deterioration	4	5%	12	15%	8	10%	12	15%

Supplement S7: ADD presentation

Table S3. Baseline Descriptive Demographics of the ADD Presentation compared to other Presentations

	ADD N = 71	Others N = 152	
	Mean (SD)	Mean (SD)	p-value [a]
Age	9.3 (1.8)	8.2 (1.9)	< .0001
IQ	98.0 (12.8)	102.4 (13.5)	0.027
Inattention problems rated by parent	1.6 (0.6)	1.5 (0.6)	0.292
Hyperactivity-impulsivity problems rated by parent	1.3 (0.9)	1.7 (0.5)	< .0001
Emotion regulation problems rated by parent	0.5 (0.1)	0.6 (0.1)	0.001
Inattention problems rated by teacher	1.5 (0.5)	1.3 (0.5)	0.125
Hyperactivity-impulsivity problems rated by teacher	1.0 (1.1)	1.5 (0.6)	< .0001
Emotion regulation problems rated by teacher	0.5 (0.1)	0.4 (0.1)	0.004
	% (N)	% (N)	*p-value* [b]
Male sex	64.8 (46)	78.3 (119)	0.032
Comorbidities			
ODD	22.4 (15)	48.0 (72)	< .0001
Probable ASD	9.0 (6)	18.7 (26)	0.070
Clinically elevated internalizing problems [a]	32.8 (22)	33.8 (50)	0.891
Treatment history			0.660
None	20.0 (14)	19.0 (28)	

Medication	20.0 (14)	21.1 (31)
Child focused psychological therapy	42.9 (30)	36.1 (53)
Parent focused therapy	17.1 (12)	23.8 (35)

Note. Others include the combined or hyperactivity-impulsivity presentation and NAO. [a] F-test; [b] Chi-square test.

To evaluate whether the criteria for the ADHD research diagnosis were appropriate to determine the ADD presentation, we compared baseline characteristics of the ADD presentation group to all other presentations (see Table S3). Table S3 includes only child characteristics, because no differences were found on parent characteristics (see Table 1 for parent characteristics). Results revealed differences between groups such as more girls and lower comorbid ODD problems in the ADD presentation group compared to the other presentations, which are in line with previous studies [19, 20].

Supplement S8: assessment of comorbidity

ODD was based on the K-SADS filled out by parents and the conduct problem subscale of the SDQ filled out by teachers. Specifically, ODD was defined by a total score on the K-SADS of eight or higher, including at least three items with a score of three (‘severe problems’), and a score of four or higher (range 0-10) on the SDQ subscale.

Probable ASD was based on the CSBQ and prosocial behavior subscale of the SDQ, both filled out by parents and teachers. Specifically, probable ASD was defined by 1) a score equal to or higher than the cut-off score on the CSBQ filled out by parents [10] or a score equal to or lower than four (range 0-10) on the SDQ subscale filled out by parents, and 2) a score equal to or higher than the cut-off score on the CSBQ filled out by teachers based on age and sex [10] or a score equal to or lower than four on the SDQ subscale filled out by teachers.

Clinically elevated internalizing problems were based on the emotional symptoms subscale of the SDQ filled out by parents and teachers. Specifically, this was defined by a score equal to or higher than five (range 0-10) of the subscale filled out by parents and/or a score equal to or higher than six of the subscale filled out by teachers.

Supplement S9: adherence to dietary treatments

Table S4. Adherence to Elimination Diet

		Adherence dietician			
		Excellent	Good	Insufficient	Total
Adherence parent	Excellent	63.5 (47)	10.8 (8)	1.4 (1)	75.7 (56)
	Good	6.8 (5)	10.8 (8)	1.4 (1)	18.9 (14)
	Insufficient	1.4 (1)	0.0 (0)	4.1 (3)	5.4 (4)
	Total	71.6 (53)	21.6 (16)	6.9 (5)	100.0 (74)

Values represent % (N)

Table S5. Adherence to Healthy Diet

		Adherence dietician			
		Excellent	Good	Insufficient	Total
Adherence parent	Excellent	61.6 (45)	2.7 (2)	2.7 (2)	67.1 (49)
	Good	6.8 (5)	16.4 (12)	2.7 (2)	26.0 (19)
	Insufficient	0.0 (0)	2.7 (2)	4.1 (3)	6.8 (5)
	Total	68.5 (50)	21.9 (16)	9.6 (7)	100.0 (73)

Values represent % (N)

Results showed a significant difference in adherence between ED participants who followed the diet until T1 and ED participants who quit the diet before T1. The latter group showed more often insufficient adherence to treatment before quitting χ^2 (2, N = 147) = 8.06, p = 0.018. For HD participants, a trend was found χ^2 (2, N = 147) = 5.29, p = 0.071. Therefore, proportions of adherence were compared in the HD group using a z-test with Bonferroni corrections. Results showed a difference of 26.2% (95% CI [3.87, 48.61]) in the category insufficient adherence: more HD participants who quit the diet before T1 were categorized in the insufficient adherence group compared to participants who followed the diet until T1.

Logistic regression analyses using the backward step method were run to determine which factors predicted good to excellent adherence to the dietary treatments. Analyses including child characteristics as predictors showed that older children were less likely to show good or excellent adherence to the diets, rated by parents (OR: 0.57, 95% CI [0.34, 0.95], p = 0.030). The interaction term with treatment was significant (OR: 0.19, 95% CI [0.04, 0.99], p = 0.037) indicating that specifically older children following the HD were less likely to show good or excellent adherence to the HD. Adherence rated by dieticians showed the same trend for age and adherence (OR: 0.69, 95%

CI [0.47, 1.01], $p = 0.059$). In addition, children with higher severity of emotion regulation problems at baseline rated by teachers were less likely to show good or excellent adherence to the diets, rated by dieticians (OR: 0.78, 95% CI [0.64, 0.94], $p = 0.009$). The interaction term with treatment was not significant (OR: 1.18, 95% CI [0.49, 2.86], $p = 0.714$).

Analyses including parental characteristics as predictors showed that children of parents with higher prior believes about success of treatment were more likely to show good or excellent adherence, rated by parents (OR: 9.65, 95% CI [1.62, 57.62], $p = 0.013$). Children of mothers with secondary level of education (see Table 1) were more likely to show good or excellent adherence, rated by parents (OR: 0.78, 95% CI [0.64, 0.94], $p = 0.017$). Moreover, children of fathers with another country of birth than the Netherlands were less likely to show good or excellent adherence, rated by parents (OR: 0.10, 95% CI [0.01, 0.60], $p = 0.012$). Adherence rated by dieticians showed the same result for country of birth of father and adherence (OR: 0.10, 95% CI [0.01, 0.32], $p = 0.001$). Interaction terms with treatment were not significant for these predictors (OR: 0.10, 95% CI [0.01, 11.62], $p = 0.343$), (OR: 3.57, 95% CI [0.05, 254.99], $p = 0.559$), (OR: 24.07, 95% CI [0.14, 4141.82], $p = 0.226$), (OR: >100, 95% CI [n.a.], $p = .997$), respectively. Finally, when parents more often used the parenting style 'punishment', a trend significant results showed that children were less likely to show good or excellent adherence, rated by dieticians (OR: 0.33, 95% CI [0.11, 0.97], $p = 0.051$). The interaction term with treatment was not significant (OR: 1.57, 95% CI [0.11, 23.42], $p = 0.742$).

Table S6. Treatment, Nutritional and Health Baseline Characteristics

	**Elimination Diet N = 84**	**Healthy Diet N = 81**	**Care As Usual N = 58**
	Mean (SD)	Mean (SD)	Mean (SD)
**Time in weeks between start treatment and T1**	5.0 (0.4)	5.0 (0.3)	5.2 (0.6)
**Total amount of dietician consults needed**	6.5 (2.0)	5.8 (1.5)	n.a.
**Participation/treatment experience (range 1-10)**	7.6 (1.6)	7.3 (1.6)	7.6 (1.6)
**Parental prior believes (range 1-4) T0** [a]	3.3 (0.5)	3.3 (0.6)	3.5 (0.7)
**Nutritional intake at T0** [b]			
Energy (kcal)	1722.0 (361.5)	1776.4 (350.5)	1600.2 (324.0)
Carbohydrates (en%)	50.1 (6.0)	49.9 (6.0)	51.7 (5.7)
Proteins (en%)	14.0 (2.3)	14.5 (2.6)	14.5 (2.7)
Total fat (en%)	33.3 (5.6)	33.0 (5.6)	31.4 (6.0)
Dietary fibre (g per mJ)	2.7 (0.7)	2.6 (0.6)	2.5 (0.6)
Sugar (en%)	24.4 (6.2)	23.1 (5.8)	24.7 (7.1)
Vitamin B12 (mug)	2.9 (1.4)	3.0 (1.4)	2.9 (1.3)
Vitamin D (mug)	2.0 (1.3)	2.2 (1.8)	1.7 (1.0)
Folic acid (mug)	188.9 (65.9)	191.4 (65.6)	164.9 (61.9)
Magnesium (mg) [c]	249.3 (67.1)	264.1 (73.2)	222.0 (65.7)
Iron (mg)	8.2 (2.6)	8.8 (3.2)	7.9 (2.6)
Calcium (mg)	730.3 (290.8)	809.0 (344.0)	688.9 (256.9)
Zinc (mg)	7.7 (2.1)	8.0 (2.3)	7.3 (1.9)
Potassium (mg)	2374.7 (566.8)	2461.2 (625.1)	2255.4 (649.7)
	hours:min (SD)	hours:min (SD)	hours:min (SD)
**Total amount of time needed for dietician consults**	5:19 (2:24)	5:05 (1:54)	n.a.

	% (N)	% (N)	% (N)
**Do parents expect a relationship between food and child behavior?**			
Yes	48.8 (41)	43.2 (35)	15.5 (9)
Maybe	42.9 (36)	54.3 (44)	39.7 (23)
No	8.3 (7)	2.5 (2)	43.1 (25)
**Consuming breakfast everyday T0**			
Yes	97.6 (82)	96.3 (78)	89.3 (50)
No	2.4 (2)	3.7 (3)	10.7 (6)
**Overweight T0** [d]	2.4 (2)	11.1 (9)	6.3 (3)
**Adverse Events**			
Increased perceived stress within family[e]	3.6 (3)	2.4 (2)	n.a.
Child resistant to the diet[f]	1.2 (1)	1.1 (1)	n.a.

*Note.*SD = Standard Deviation; en% = energy percent; g = grams; n.a. = not applicable. [a] higher scores reflect higher believes in successful effects of treatment; [b] a complete overview of all assessed micronutrients can be found in Appendix S10; [c] no significant difference after correcting for energy (kcal); [d] based on international cut off points for BMI for overweight [21]; [e] After one week, three and four weeks of the Elimination Diet and after two weeks in the Healthy Diet; [f] after three and five weeks in the Elimination Diet and after five days in the Healthy Diet.

Supplement S10: micronutrient intake

Table S7. Micronutrient Intake at Baseline

Micronutrients	Elimination Diet	Healthy Diet	Care As Usual
	Mean (SD)	Mean (SD)	Mean (SD)
Sodium (mg)	1941.3 (623.9)	2067.3 (611.8)	1824.8 (456.5)
Salt (g)	4.9 (1.6)	5.2 (1.5)	4.6 (1.1)
Phosphorus (mg)	1080.0 (283.2)	1152.7 (317.8)	1011.8 (277.5)
Selenium (mug)	31.5 (11.4)	33.1 (11.8)	31.4 (9.1)
Iodine (mug)	152.3 (66.7)	167.0 (48.1)	144.0 (43.1)
Vitamin A (mug)	611.2 (452.1)	585.9 (374.5)	567.3 (405.4)
Vitamin B1 (mg)	0.8 (0.3)	0.8 (0.3)	0.7 (0.3)
Vitamin B2 (mg)	1.1 (0.4)	1.2 (0.5)	1.1 (0.5)
Vitamin B6 (mg)	1.1 (0.5)	1.2 (0.5)	1.2 (0.5)
Vitamin C (mg)	79.2 (39.9)	73.9 (39.1)	86.3 (49.4)
Vitamin E (mg)	9.0 (4.8)	9.9 (4.2)	9.5 (4.3)

CAU participants had lower phosphorus intake compared to HD participants ($p = 0.042$), which was non-significant after correcting for energy intake ($p = 0.326$).

Supplement S11: assumptions statistical analyses

Most assumptions of the cumulative odds ordinal logistic regression were met: there were proportional odds, as assessed by a full likelihood ratio test comparing the fitted model to a model with varying location parameters, $\chi^2(3) = 5.53$, $p = 0.137$. The final model did not significantly predict the dependent variable over and above the intercept-only model, $\chi^2(1) = 1.82$ $p = .18$. In addition, the deviance goodness-of-fit test indicated that the model was a good fit to the observed data, $\chi^2(3) = 5.53$, $p = 0.137$.

Most assumptions of ANCOVA were met: there was no significant interaction between the treatment arm and any of the T0 variables, suggesting that the assumption of homogeneity of the regression slopes was met. The Levene F test was not significant for most dependent variables (except for two), which also confirms the homogeneity of regression. For BMI ($p = 0.001$) and parent rated ER ($p = 0.036$) this assumption was violated. However, ANCOVA is quite robust when this assumption is violated, if sample sizes do not differ from each other by more than a factor of three. This is the case in the present study with group sample sizes of 84, 81 and 58. In addition, the two dietary treatments did not differ on baseline ADHD and emotion regulation problems (Table 3). Standardized residuals for the interventions and for the overall model were normally distributed for the majority of variables, as assessed by Shapiro-Wilk's ($p > .05$). For diastolic and systolic blood pressure, heart rate, parental quality of life, somatic complaints and positive parental engagement, this assumption was violated. Inspecting histograms, skewness and kurtosis values resulted for positive engagement, parental quality of life and somatic complaints in no significant values for skewness or kurtosis. Transforming these variables did not improve normality. Given the fact that ANCOVA is fairly robust when this assumption is violated, we did not transform the variables and used the original variables in the analyses. Using a Van der Waerden transformation for blood pressure and heart rate did improve normality in these variables. However, clinical interpretation deteriorated when using this transformation. Given this fact and given that ANCOVA is fairly robust for non-normally distributed data, we did not transform these variables.

Supplement S12: comparisons of proportions of respondership

Tables S8 through S13 show results of comparisons of proportions of respondership between the treatment groups post-hoc per category using a z-test with Bonferroni corrections (these provide confidence intervals without exact *p*-values).

Table S8. Post-hoc Comparisons between Categories of Respondership Elimination Diet versus Healthy Diet

	Elimination Diet (N = 84)		Healthy Diet (N = 81)	
	n	%	n	%
Full responders	9	10.7%	14	17.3%
Partial responders	20	23.8%	27	33.3%
Mixed responders *	38	45.2%	21	25.9%
Non-responders	6	7.1%	8	9.9%
Deterioration	11	13.1%	11	13.6%

Note. * represents a significant difference of 19.3 % (95% CI [4.68, 33.94], p <.05)

Table S9. Post-hoc Comparisons between Categories of Respondership ED versus CAU

	Elimination Diet (N = 84)		CAU (N=58)	
	n	%	n	%
Full responders *	9	10.7%	18	31.6%
Partial responders	20	23.8%	14	24.6%
Mixed responders	38	45.2%	17	29.8%
Non-responders	6	7.1%	3	5.3%
Deterioration	11	13.1%	5	8.8%

Note. * represents a significant difference of 20.9% (95% CI [7.63, 34.10], p < .05)

Table S10. Post-hoc Comparisons between Categories of Respondership HD versus CAU

	Healthy Diet (N=81)		CAU (N=58)	
	n	%	n	%
Full responders	14	17.3%	18	31.6%
Partial responders	27	33.3%	14	24.6%
Mixed responders	21	25.9%	17	29.8%
Non-responders	8	9.9%	3	5.3%
Deterioration	11	13.6%	5	8.8%

Table S11. Post-hoc Comparisons between Categories of Respondership with Full and Partial Responders combined ED versus HD

	Elimination Diet (N = 84)		Healthy Diet (N = 81)	
	n	%	n	%
Full and partial responders *	29	34.5%	41	50.6%
Mixed responders *	38	45.2%	21	25.9%
Non-responders	6	7.1%	8	9.9%
Deterioration	11	13.1%	11	13.6%

Note. * represents a significant difference of 16.1% (95% CI [1.01, 21.18], *p* <.05) and 19.3 % (95% CI [4.68, 33.94], *p* <.05) respectively

Table S12. Post-hoc Comparisons between Categories of Respondership with Full and Partial Responders combined ED versus CAU

	Elimination Diet (N = 84)		CAU (N=58)	
	n	%	n	%
Full and partial responders	29	34.5%	32	56.1%
Mixed responders	38	45.2%	17	29.8%
Non-responders	6	7.1%	3	5.3%
Deterioration	11	13.1%	5	8.8%

Note. * represents a significant difference of 21.6% (95% CI [4.95, 38.28], $p < .05$)

Table S13. Post-hoc Comparisons between Categories of Respondership with Full and Partial Responders combined HD versus CAU

	Healthy Diet (N=81)		CAU (N=58)	
	n	%	n	%
Full and partial responders	41	50.6%	32	56.1%
Mixed responders	21	25.9%	17	29.8%
Non-responders	8	9.9%	3	5.3%
Deterioration	11	13.6%	5	8.8%

Supplement 13: percentage change in T0 versus T1

Figure S1. Percentage Change in T0 versus T1 in ADHD and ER Problems per Respondership Category

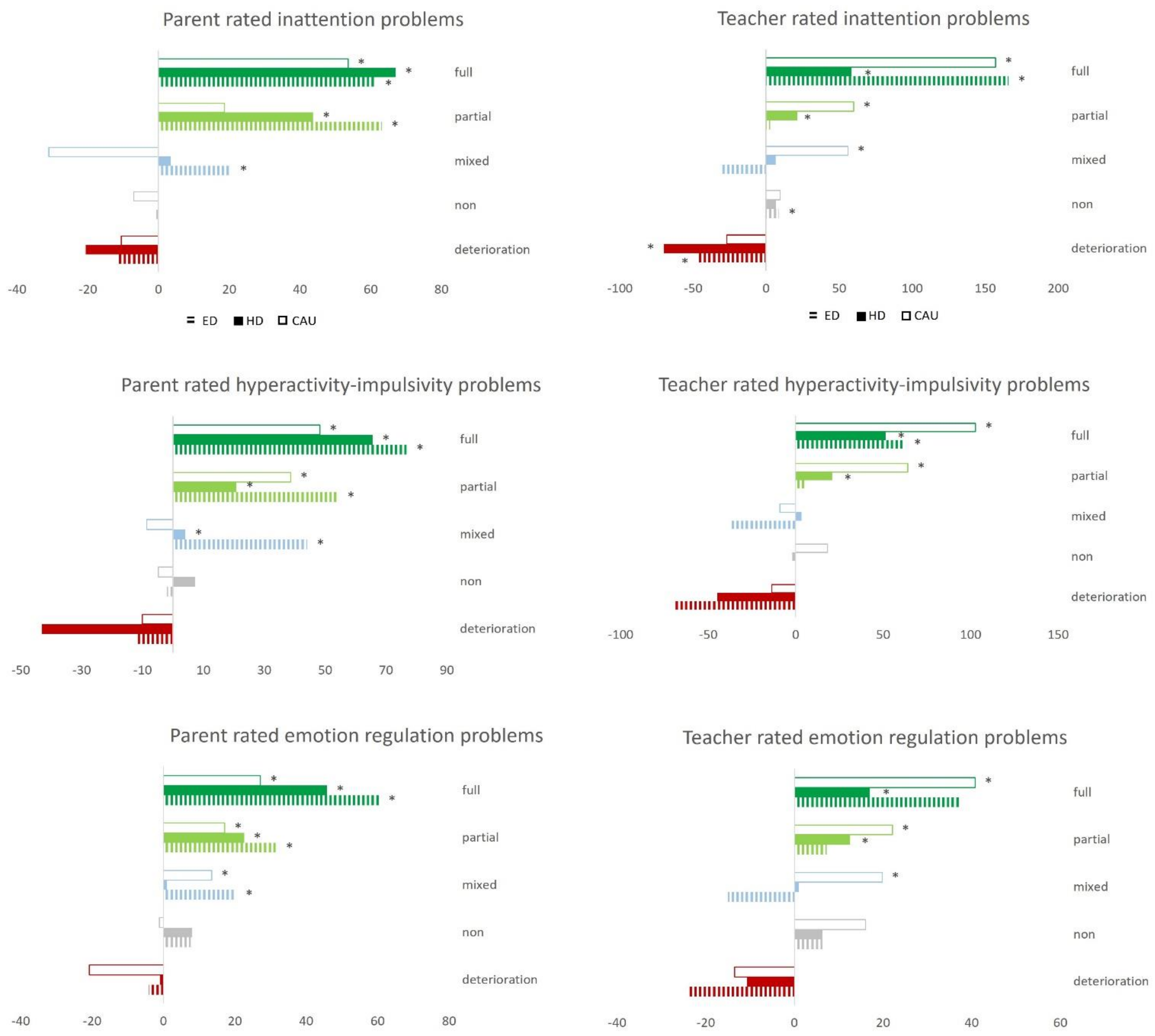

Note. * *p* <.05; H/I = hyperactivity-impulsivity problems; ER = emotion regulation problems; Parent = rated by parent; Teacher = rated by teacher

Figure S1 illustrates that in the CAU group teachers observe an overall stronger response to treatment compared to parents, whereas in the dietary treatments this pattern was reversed. Significant deterioration of symptoms was only observed in the dietary treatments based on teacher reported ADHD behaviors. In the partial responder group, improvement in behaviors at school and at home was

found for children receiving HD or CAU; for children improving partially after receiving ED, this was completely attributable to parental ratings. In addition, compared to HD and CAU, the mixed responders in the ED group seem to consist more often of parents who report improvement and teachers who report deterioration.

Supplement S14: predicting specific respondership categories

Table S14. Logistic Regression Analyses using Baseline Measurements to predict specific Respondership Categories versus all other Respondership Categories for Dietary Treatments

	Inattention problems rated by parents[a]	Inattention problems rated by teacher[a]	Internalizing problems rated by parent[a]	Emotion dysregulation problems rated by teacher[a]	Parental quality of life[a]	ADD presentation[b]	Secundary education mother	Country of birth mother other than Netherlands
	Mean (SD)	Mean (SD)	Mean (SD)	Mean (SD)	Mean (SD)	% (N)	% (N)	% (N)
**Full responders vs. others**	13.5 (5.1) vs. 12.7 (6.5)	10.3 (4.7) vs. 12.4 (6.7)	3.9 (2.9) vs. 3.1 (2.3) **1.25 (1.02, 1.53) *p* = 0.029**	11.9 (3.4) vs. 12.9 (4.6)	1.5 (0.4) vs. 1.6 (0.4)	29.3 (12) vs. 32.6 (50)	78.3 (18) vs. 80.3 (114)	4.3 (1) vs. 6.3 (9)
**Partial responders vs. others**	13.0 (5.8) vs. 12.7 (6.5)	14.9 (6.2) vs. 11.0 (6.3) **1.09 (1.1, 1.17) *p* = 0.029**	3.0 (2.2) vs. 3.3 (2.5)	15.0 (4.4) vs. 11.8 (4.2) **1.16 (1.04, 1.29) *p* = 0.006**	1.6 (0.4) vs. 1.6 (0.4)	24.6 (15) vs. 34.8 (56)	83 (39) vs. 78.8 (93)	2.1 (1) vs. 7.6 (9)
**Mixed responders vs. others**	10.9 (7.2) vs. 13.8 (5.5) **0.93 (0.87, 1.00) *p* =0.043**	10.1 (6.3) vs. 13.2 (6.4)	3.1 (2.4) vs. 3.3 (2.5)	10.6 (4.2) vs. 13.9 (4.2) **0.77 (0.67, 0.88) *p* < .0001**	1.6 (0.3) vs.1.6 (0.4)	42.1 (32) vs. 26.7 (39) **1.97 (1.10, 3.56) *p* = 0.024**	71.2 (42) vs. 84.9 (90) **3.38 (1.28, 8.90) *p* = 0.014**	11.9 (7) vs. 2.8 (3) **10.88 (1.56, 75.97) *p* = 0.016**
**Non-responders vs. others**	16.0 (4.3) vs. 12.5 (6.3)	17.3 (3.8) vs. 11.6 (6.5) **1.21 (1.03, 1.43) *p* = 0.022**	2.8 (2.5) vs. 3.3 (2.4)	15.8 (3.7) vs. 12.5 (4.5)	1.7 (0.3) vs. 1.6 (0.4) **7.62 (1.08, 53.69) *p* = 0.041**	11.8 (2) vs. 33.7 (69)	100 (14) vs. 78.1 (118)	0.0 (0) vs. 6.6 (10)
**Deterioration vs. others**	14.7 (5.6) vs. 12.5 (6.3)	10.5 (7.4) vs. 12.3 (6.4)	3.5 (2.5) vs. 3.2 (2.4)	12.8 (3.8) vs. 12.7 (4.6)	1.8 (0.4) vs. 1.6 (0.4)	37.0 (10) vs. 31.3 (61)	86.4 (19) vs. 21.0 (30)	4.5 (1) vs. 6.3 (9)

Note. Full; partial; mixed; non; deterioration is coded as 1 and category others is coded as 0; bold numbers depict significant odds ratio (confidence interval). [a] higher scores reflect more problems or lower parental quality of life; [b] ADD presentation is coded as 1 and other presentations as 0.

Table S13 displays only the baseline characteristics where a significant predictive effect was found. Results demonstrate that more inattention problems at baseline predicted higher chances of partial and non-respondership and lower chances of mixed respondership. Moreover, more emotion regulation problems (rated by teacher) at baseline predicted higher odds of partial respondership and lower odds of mixed respondership. ADD presentation predicted higher odds of mixed respondership. More internalizing problems (rated by parent) at baseline predicted higher odds of full respondership. Lower parental quality of life at baseline predicted higher odds of non-respondership. Finally, children of mothers with secondary education (i.e. junior general secondary, senior secondary vocational, senior general secondary, pre-university) and mothers with another country of birth than the Netherlands had higher odds of being categorized as mixed responders.

Supplement S15: results of ANCOVA of secondary outcomes

Table S15. Results of ANCOVA of Secondary Outcomes

		Elimination Diet N=84				Healthy Diet N=81				Care as usual N=58				Between-group differences T0	Between-group differences T1	
	N total[a]	T0	T1	T0-T1		T0	T1		T0-T1	T0	T1	T0-T1				
		Mean (SD)	Mean (SD)	d	p-value	Mean (SD)	Mean (SD)	d	p-value	Mean (SD)	Mean (SD)	d	p-value	p-value	p-value	η_p^2
**BMI-SDS[b]**	212/208	0.3 (1.0)	0.04 (0.9)	0.79	< .0001	0.5 (1.2)	0.3 (1.1)	0.89	<.0001	0.3 (1.1)	0.2 (1.1)	0.29	0.062	0.383	0.056	0.04
**Heart rate**	206/204	79.1 (10.6)	74.9 (11.5)	0.39	0.001	78.4 (11.4)	74.2 (9.8)	0.39	0.001	78.6 (9.1)	86.4 (10.7)	-0.73	<.0001	0.916	ED<CAU: <.0001 HD<CAU: <.0001	ED<CAU: 0.23 HD<CAU: 0.29
**Systolic blood pressure**	206/204	102.7 (10.8)	99.5 (9.5)	0.34	0.006	103.5 (8.6)	98.9 (9.0)	0.43	< .0001	102.3 (10.0)	103.2 (8.8)	-0.11	0.472	0.770	ED<CAU: 0.005 HD<CAU: 0.003	ED<CAU: 0.07 HD<CAU: 0.07
**Diastolic blood pressure**	206/204	63.4 (9.7)	61.2 (7.8)	0.27	0.021	64.3 (8.6)	61.2 (7.4)	0.29	0.016	61.3 (6.7)	65.0 (5.0)	-0.56	0.002	0.188	ED<CAU: <.0001 HD<CAU: 0.001	ED<CAU: 0.13 HD<CAU: 0.10
**Somatic complaints (range 1-4)[c]**	222/220	1.5 (0.3)	1.4 (0.3)	0.39	0.005	1.4 (0.3)	1.3 (0.3)	0.24	0.043	1.4 (0.3)	1.5 (0.3)	-0.05	0.694	0.359	ED<CAU: 0.016 HD<CAU: 0.024	ED<CAU: 0.04 HD<CAU:0.04
**Parental stress (range 1-4)[c]**	221/220	1.8 (0.4)	1.6 (0.4)	0.61	< .0001	1.7 (0.4)	1.6 (0.4)	0.52	< .0001	1.7 (0.3)	1.6 (0.3)	0.30	0.041	0.243	0.410	
**Parenting style (range 1-5)[d,e]**	174/169															

**-Positive engagement**		4.3 (0.4)	4.3 (0.4)	-0.05	0.969	4.3 (0.5)	4.3 (0.5)	0.004	0.969	n.a.	n.a.	n.a.	n.a.	0.521	0.969	0.002
**-Punishment**		3.1 (0.8)	3.0 (0.8)	0.11	0.927	3.3 (0.7)	3.2 (0.7)	0.23	0.306	n.a.	n.a.	n.a.	n.a.	0.053	0.969	0.000
**Quality of life parent (range 1-3)[e,f]**	175/171	1.6 (0.4)	1.5 (0.3)	0.47	< .0001	1.6 (0.4)	1.5 (0.4)	0.49	< .0001	n.a.	n.a.	n.a.	n.a.	0.662	0.600	0.002
**Parental happiness (range 0-10)[e,g]**	175/170	7.2 (1.3)	7.6 (1.1)	-0.35	0.006	7.1 (1.4)	7.4 (1.4)	-0.24	0.057	n.a.	n.a.	n.a.	n.a.	0.615	0.297	0.007
**Family functioning (range 1-4)[c,e]**	174/170	3.3 (0.4)	3.3 (0.4)	0.03	0.811	3.2 (0.4)	3.3 (0.4)	0.06	0.811	n.a.	n.a.	n.a.	n.a.	0.181	0.811	0.003
		% (N)	% (N)			% (N)	% (N)			% (N)	% (N)					
**Sleep problems**	221/218			n.a.	< .0001			n.a.	0.99			n.a.	0.656	0.367	ED<CAU: 0.002<br>ED<HD: <0.0001	n.a.
**-None**		44.0 (37)	71.4 (60)			53.1 (43)	51.9 (42)			55.2 (32)	46.6 (27)					
**-Problems falling asleep or maintaining sleep**		54.8 (46)	27.4 (23)			45.7 (37)	45.7 (37)			44.8 (26)	50.0 (29)					

Note. d = Cohen's d; η_p^2 = partial eta squared; n.a. = not applicable. [a] values represent N=T0/N=T1; [b] SDS = Standard Deviation Score (how many SD's does a measure deviate from the median); [c] higher scores reflect more problems; [d] higher scores reflect more engagement in this parenting style; [e] not applicable for CAU group, because parents did not fill out this questionnaire; [f] higher scores reflect lower quality of life; [g] higher scores reflect higher happiness

Supplement S16: different parental raters at T0 and T1

For 17 participants (N = 6 ED participants, N = 9 HD participants; N = 2 CAU participants), mothers filled out questionnaires at T0 and fathers at T1, or vice versa. A sensitivity analysis without these participants was performed, to examine if the results of the ordinal regression analysis changed. Results showed the same pattern and a trend significant result was found: the odds ratio of being in a better response category for ED participants versus HD participants was 0.59, 95% CI [0.33, 1.11], p = 0.078.

Running the ordinal regression analysis without these 17 participants, showed the same pattern when comparing the ED to the CAU group: the odds ratio of being in a better response category for ED participants versus the CAU group was 0.39, 95% CI [0.20, 0.73], p = 0.004. The odds ratio of being in a better response category for HD participants versus the CAU group also showed the same pattern: 0.66, 95% CI [0.35, 1.24], p = 0.196.

Results of secondary outcome measurements also showed the same pattern when this subsample of participants was excluded from the analyses. All in all, the results of almost all sensitivity analyses did not differ from the original analyses, therefore we chose to include all participants while running the analyses.

Supplement S17: influence Covid-19

A total of 14 participants started the diet right after the first lockdown due to the Covid-19 pandemic in the Netherlands in May 2020. These participants did go to school again, but schooldays were only half of the time face-to-face which might have made it more difficult for teachers to rate the behavior of the participants. A sensitivity analysis without these participants was performed, to examine if the results of the ordinal regression analysis changed. Results showed the same pattern and a trend significant result was found: the odds ratio of being in a better response category for ED participants versus HD participants was 0.58, 95% CI [0.33, 1.0], $p = 0.066$. In addition, the distribution of respondership remained the same without these participants: ED participants were still significantly more often categorized as mixed responders.

The screening (i.e. K-SADS and SWAN) of the 14 participants already took place three to four months prior to T0, because the T0 had to be moved to after the lockdown due to school closure (to prevent missing teacher ratings in the primary outcome data). The time between screening and T0 for the other participants usually was shorter (e.g. one month). Consequently, it could be that these 14 participants might show less or more ADHD problems compared to the screening three to four months ago. Therefore, independent t-tests were run to examine if T0 scores on in attention, hyperactivity-impulsivity and emotion dysregulation of participants who did not start the diet after the first lockdown differed from T0 scores of the 14 participants who did start the diet right after the first lockdown. No differences were found for parent rated inattention ($t(161) = -1.04$, $p = 0.301$), hyperactivity-impulsivity ($t(161) = -\ 0.35$, $p = 0.724$), and emotion dysregulation ($t(161) = -0.70$, $p = 0.491$), and for teacher inattention ($t(159) = 0.85$, $p = 0.397$), hyperactivity-impulsivity ($t(159) = -0.26$, $p = 0.792$), and emotion dysregulation ($t(19.11) = -0.68$, $p = 0.628$).

References

1. Reichart CG, Wals M, Hillegers M (2000) Nederlandstalige versie van de Kiddie-SADS. Utrecht: H.G. Rümke Groep.
2. Swanson JM et al 2017 Young adult outcomes in the follow-up of the multimodal treatment study of attention-deficit/hyperactivity disorder: symptom persistence, source discrepancy, and height suppression. J Child Psychol Psychiatry 58(6):663-678. https://doi.org/10.1111/jcpp.12684.
3. Bosch A et al 2020 A two arm randomized controlled trial comparing the short and long term effects of an elimination diet and a healthy diet in children with ADHD (TRACE study). Rationale, study design and methods. BMC Psychiatry 20(1):262. https://doi.org/10.1186/s12888-020-02576-2.
4. Wechsler D (2002) WISC-III NL. Handleiding. London: Psychological Corporation Limited.
5. Pelsser LM et al 2011 Effects of a restricted elimination diet on the behaviour of children with attention-deficit hyperactivity disorder (INCA study): A randomised controlled trial. Lancet 377(9764):494-503. https://doi.org/10.1016/S0140-6736(10)62227-1.
6. Willmott SA, Boardman JA, Henshaw CA, Jones PW 2004 Understanding General Health Questionnaire (GHQ-28) score and its threshold. Soc Psychiatry Psychiatr Epidemiol 39(8):613-7. https://doi.org/10.1007/s00127-004-0801-1.
7. Roede MJ, Van Wieringen JC 1985 Growth diagrams 1980: Netherlands third nation-wide survey. Tijdschr Soc Gezondheidsz 63:1-34.
8. Schonbeck Y, Van Buuren S 2010 Factsheet Resultaten Vijfde Landelijke Groeistudie. Leiden: TNO.
9. Pelham WE 1993 Pharmocotherapy for children with attention-deficit hyperactiviy disorder. School Psych Rev 22:199-227.
10. Hartman CA, Luteijn E, Moorlag A, De Bildt A, Minderaa R (2007) Handleiding voor de VISK [Manual for the CSBQ]. Amsterdam, the Netherlands: Boom Uitgevers.
11. Goodman R 1997 The Strengths and Difficulties Questionnaire: a research note. J Child Psychol Psychiatry 38(5):581-586.
12. Veerman JW et al (2012) Vragenlijst Gezinsfunctioneren volgens Ouders (VGFO): Handleiding. Nijmegen, the Netherlands: Praktikon.
13. Van Leeuwen KG et al (2013) Verkorte Schaal voor Ouderlijk Gedrag (VSOG). Nijmegen, the Netherlands: Praktikon.
14. Brouwer WB, van Exel NJ, van Gorp B, Redekop WK 2006 The CarerQol instrument: a new instrument to measure care-related quality of life of informal caregivers for use in economic evaluations. Qual Life Res 15(6):1005-21. https://doi.org/10.1007/s11136-005-5994-6.
15. Vermulst A, Kroes G, De Meyer R, Nguyen L, Veerman JW (2011) Opvoedingsbelasting vragenlijst - versie voor ouders en jeugdigen van 0 t/m 18 jaar. Nijmegen, the Netherlands: Praktikon.
16. Ocke M, Dinnissen C, Stafleu A, de Vries J, van Rossum C 2021 Relative Validity of MijnEetmeter: A Food Diary App for Self-Monitoring of Dietary Intake. Nutrients 13(4). https://doi.org/10.3390/nu13041135.
17. NEVO. S 2011 NEVO-tabel. Nederlands Voedingsstoffenbestand. Den Haag: Voedingscentrum.
18. Johnstone JM et al 2020 Development of a Composite Primary Outcome Score for Children with Attention-Deficit/Hyperactivity Disorder and Emotional Dysregulation. J Child Adolesc Psychopharmacol 30(3):166-172. https://doi.org/10.1089/cap.2019.0179.

19. Milich R, Balentine AC, Lynam DR 2001 ADHD Combined Type and ADHD Predominantly Inattentive Type Are Distinct and and unrelated disorders. Clin Psychol: Sci Pract 8(4):463.
20. Solanto MV, Pope-Boyd SA, Tryon WW, Stepak B 2009 Social functioning in predominantly inattentive and combined subtypes of children with ADHD. J Atten Disord 13(1):27-35. https://doi.org/10.1177/1087054708320403.
21. Cole TJ, Bellizzi MC, Flegal KM, Dietz WH 2000 Establishing a standard definition for child overweight and obesity worldwide: international survey. BMJ 320(7244):1240.
